# Noninvasive Interstitial Glucose Estimation Using Wearables and Machine Learning in Healthy Individuals and Individuals With Obesity: Observational Cohort Study

**DOI:** 10.2196/91724

**Published:** 2026-09-30

**Authors:** Franziska Schmelter, Xinyu Huang, Annika Heidmann, Paul Beier, Friederike Miriam Rundfeldt, Christina Plöger, Artur Piet, Md Abid Hasan, Yuanheng Zhang, Lennart Jablonski, Oliver Witt, Torsten Schröder, Stefanie Derer, Chris Easthope Awai, Marcin Grzegorzek, Christian Sina

**Affiliations:** 1 Institute of Nutritional Medicine, University of Luebeck, Ratzeburger Allee 160, Luebeck, Schleswig-Holstein, Germany, 49 451 3101 ext 8422; 2 University Hospital Schleswig-Holstein, Luebeck, Schleswig-Holstein, Germany; 3 expandAI GmbH, Luebeck, Schleswig-Holstein, Germany; 4 Institute of Medical Informatics, University of Luebeck, Luebeck, Schleswig-Holstein, Germany; 5 German Research Center for Artificial Intelligence (DFKI), Luebeck, Schleswig-Holstein, Germany; 6 Research & Development, Perfood Laboratories GmbH, Luebeck, Schleswig-Holstein, Germany; 7 Data Analytics & Rehabilitation Technology (DART), Lake Lucerne Institute, Vitznau, Switzerland; 8 Khalifa University, Abu Dhabi, United Arab Emirates

**Keywords:** continuous glucose monitoring, wearables, interstitial glucose, machine learning algorithm, personalized nutrition

## Abstract

**Background:**

Continuous glucose monitoring (CGM) can facilitate weight management and lower the risk of metabolic diseases by providing real-time feedback on glycemic responses, thereby enabling more informed lifestyle decisions. However, current CGM systems remain constrained by invasiveness, cost, and short sensor lifespan, limiting their practicality for guiding individualized postprandial low-glycemic diets.

**Objective:**

Extending earlier proof-of-concept findings, this study aimed to validate an interstitial glucose (IG) machine learning algorithm in real-world environments using multimodal, continuous data collected from wearable sensors and smartwatches. In the long term, we aim to embed a noninvasive, sensor-based algorithm for estimating tissue glucose within mobile health apps and postprandial low-glycemic diet frameworks to enable scalable, personalized prevention strategies.

**Methods:**

We conducted a 2-week study phase during which participants continuously wore 2 noninvasive sensor devices: a scientific sensor wristband (Empatica EmbracePlus) and a commercially available smartwatch (Fitbit Sense 2). As a reference measurement, an invasive CGM sensor (Abbott FreeStyle Libre 3) measured IG levels. For metabolic characterization, 1-point fasting blood and urine samples were collected, and deep phenotyping using state-of-the-art nuclear magnetic resonance spectroscopy and bioelectrical impedance analysis was performed. For participants with overweight and obesity, clinical standard parameters focusing on glucose metabolism were analyzed.

**Results:**

A total of 74 participants, 34 (46%) healthy controls and 40 (54%) metabolically at-risk (MR) individuals, simultaneously used an invasive CGM device together with 2 noninvasive wristbands over 2 weeks. Healthy controls were characterized by a mean age of 24.53 (SD 3.68) years and a mean BMI of 22.36 (SD 2.16) kg/m². In contrast, the MR cohort had a mean age of 55.38 (SD 15.08) years and a mean BMI of 35.38 (SD 4.91) kg/m². Furthermore, the MR cohort showed elevated fasting glucose levels (mean 107.56, SD 19.26 mg/dL), hemoglobin A_1c_ levels (mean 5.67%, SD 0.63%), and an increased homeostatic model assessment of insulin resistance index (mean 4.34, SD 3.49), indicating a disturbed glucose metabolism. The proposed long short-term memory network based on feature vectors obtained the best IG prediction performance, with an average root-mean-squared error of 21.04 (SD 8.32) mg/dL and 98.3% of predictions in zones A and B of the Clarke error grid analysis, representing a high level of predictive accuracy. In addition, based on data from a smartwatch, we achieved a comparable average root-mean-squared error of 23.49 (SD 11.46) mg/dL for the overall cohort.

**Conclusions:**

This study demonstrates that IG levels can be predicted from multimodal, noninvasive wearable sensor data using a machine learning approach under real-world conditions. While further validation in larger and more diverse cohorts is warranted, this approach represents a promising step toward accessible, personalized glycemic monitoring and dietary guidance as a preventive tool in mobile health apps.

## Introduction

Maintaining glucose homeostasis is beneficial not only for patients with type 2 diabetes (T2D) but also for healthy individuals. Stable blood glucose (BG) levels are crucial for body weight control and preventing risks such as metaflammation, insulin resistance, and associated conditions such as cardiovascular diseases, cancer, acne, migraine, and neurodegenerative diseases, indicating a wide range of applications [1-3].

A high postprandial glucose response (PPGR) can lead to postprandial hyperinsulinemia, glucotoxicity, lipogenesis, and visceral obesity [4]. Although diet is a major determinant of PPGR, standardized dietary recommendations frequently fail in practice, as eating behavior is strongly influenced by individual preferences, taste, cultural patterns, and daily routines. These factors make dietary responses and adherence highly variable. Personalized nutrition addresses this challenge by tailoring guidance to each individual’s unique habits and preferences rather than imposing a uniform diet. Previous studies have shown promising results for personalized diets in avoiding glucose spikes, thereby addressing the individual nature of PPGRs [5,6]. As an example, digital postprandial low-glycemic diets (PLGD) based on continuous glucose monitoring (CGM) have shown effectiveness for individuals with prediabetes or diabetes and migraine [7,8].

While CGM is commonly used to measure interstitial glucose (IG) levels [9], it has limitations, including invasiveness, inconvenience, high cost, limited sensor lifespan, and environmental impact [10]. Noninvasive sensor technologies offer a potential solution for real-time calculation of glucose levels through longitudinal monitoring of physiological parameters, thereby providing a foundation of individualized, adaptive, and dynamic day-to-day recommendations. Early research has examined the influence of physiological parameters on food intake and the associated development of obesity [11]. Some approaches focus on the development of noninvasive continuous glucose monitors based on optoacoustic signals [12] or microwave technology [13,14], while others seek to use existing sensor systems. Nowadays, a wide variety of wearable devices capable of continuously monitoring diverse biosignals are commonly used in everyday life (eg, smartphones, smartwatches, and fitness trackers). When combined with modern machine learning (ML) algorithms, these technologies offer the potential to elucidate relationships between physiological parameters and food intake [15], thereby enabling personalized dietary guidance. Initial data demonstrate the ability to predict IG values in real time using physiological parameters and dietary information [16-18]. However, these algorithms depend on the manual entry of daily dietary information, a process that demands considerable user adherence and is inherently time-consuming, subjective, and susceptible to error. In prior research, we demonstrated the feasibility of predicting IG levels using data from noninvasive sensors coupled with ML algorithms without using food logs. This approach yielded robust results in young, healthy individuals when assessed in controlled settings using standardized test meals, including oral glucose tolerance tests and mixed-meal tests [19].

Given the current constraints of invasive CGM and food diary–based PPGR prediction models, there is a significant opportunity to expand the availability of digital PLGD solutions. Particularly in the context of mobile health (mHealth), calculating BG levels based on noninvasive sensor data using ML algorithms represents a significant milestone. The availability of wearables such as smartwatches, smart rings, and wristbands is steadily increasing, and using these data, BG levels and trends could be calculated in the future. In combination with low-level, user-friendly documentation tools based on photos and voice recordings, individual dietary habits could be easily tracked. A corresponding mHealth tool could then provide PLGD recommendations based on individual predictions of food reactions. This scalable mHealth tool could make PLGD accessible to the global population and, therefore, become a key application for prevention and health management in the future.

To establish the foundation of this mHealth app, address the challenges of current CGM devices, and adapt our algorithm to realistic, real-world use, we conducted a 2-week study under everyday conditions with a habitual diet. During this study, we analyzed CGM data of both healthy individuals and metabolically at-risk (MR) individuals (with overweight or obesity). We included MR participants with disturbances in glucose metabolism and insulin resistance, potentially generating aberrant IG signals, to challenge our algorithm and evaluate its performance with more complex data. For noninvasive data collection, we used 2 devices: a clinically designed sensor wristband and a commercially available smartwatch. The study had 3 aims. First, we evaluated the ML algorithm to calculate IG values under real-world conditions involving complex, mixed meals. Second, we examined its generalizability across healthy and MR individuals based on the assumption that insulin resistance may alter physiological signals and reduce predictive accuracy. Third, we assessed device independence by implementing a proof-of-concept version on a commercially available smartwatch to determine whether the algorithm maintains its performance across different wearable platforms.

## Methods

### Ethical Considerations

The study examined healthy adults and adults with overweight. The study was approved by the Ethics Committee of the University of Luebeck (Az 2022‐550 and 2024‐363) according to Good Clinical Practice. Potential participants were recruited through posters, social media, and newspaper articles in northern Germany. The inclusion and exclusion criteria were deliberately broad, encompassing adults with sufficient German language proficiency to comprehend the study materials and able to operate a smartphone. Participants in the MR cohort were either overweight with at least one known metabolic comorbidity, such as hypertension, dyslipidemia, atherosclerosis, insulin resistance, gout, stroke, or heart failure, or classified as obese. All participants provided written informed consent prior to their inclusion in the study. As part of the consent process, the participants were informed about the data protection measures, the voluntary nature of study participation, and their right to withdraw from the study at any time without giving a reason and without any disadvantages. In accordance with applicable data protection regulations, the participants’ data were deidentified. The data were analyzed in pseudonymized form solely for the purposes of the study. No compensation was paid for participating in this study.

### Study Cohort and Design

In total, 74 adults (women: n=54, 73%; and men: n=20, 27%), aged between 19 and 84 years, participated in this pilot study using an observational cohort study design. Participants were asked to wear 2 sensor wristbands as well as a CGM sensor on the upper arm continuously for 14 days. Otherwise, participants were expected to behave as usual and received no further instructions other than being asked to charge their wristbands regularly. During a clinic visit, blood and urine samples were collected, and a bioelectrical impedance analysis (BIA) measurement was performed.

In more detail, noninvasive wearable data from a scientific high-end device (Empatica EmbracePlus) and a commercially available smartwatch (Fitbit Sense 2) as well as IG data from an invasive CGM device (Abbott FreeStyle Libre 3) were collected over 14 days. First, Food and Drug Administration–confirmed noninvasive Empatica EmbracePlus measured skin temperature (STEMP) at 1 Hz, electrodermal activity (EDA) at 4 Hz, triaxial acceleration (taACC) at 32 Hz, sleep assessment (Sleep) at 0.017 Hz, and photoplethysmography (PPG), which produces a blood volume pulse (BVP) to calculate the interbeat interval in seconds and the pulse rate (Plr=60 seconds/interbeat interval) at 0.017 Hz. Second, Fitbit Sense 2 (Fitbit) quantified Plr at 0.2 Hz. Third, as reference values for our algorithm, continuous IG levels were measured by Food and Drug Administration–approved CGM devices every 5 minutes with a mean absolute relative difference of 7.8% [20]. In addition, participants kept a self-guided food diary with the help of an app (Perfood) to document their normal eating habits, activity, and sleep. However, these data were not included in this study, as the primary objective was to develop and optimize the algorithm itself, while the derivation of personalized nutritional recommendations will be addressed in a subsequent step in the future. Furthermore, study participants were deeply phenotyped. First, body composition was determined using BIA (Seca). Second, 1-point fasting blood and urine samples were collected to analyze individual metabolic profiles using nuclear magnetic resonance (NMR) spectroscopy. Third, for MR participants, characterized by overweight and obesity, clinical standard parameters were quantified, including complete blood count, lipid profiles, and liver parameters (glutamate-oxaloacetate transaminase, glutamate-pyruvate transaminase, and gamma-glutamyl transferase). Furthermore, glucose metabolism was characterized in detail by analyzing fasting glucose, insulin, homeostatic model assessment of insulin resistance, and hemoglobin A_1c_ (HbA_1c_) levels to identify potential disorders indicating a prediabetic or diabetic status of the individuals.

### NMR Spectroscopy

Serum samples were thawed at room temperature and prepared according to the standard operating procedure described by Dona et al [21]. In brief, samples were homogenized 1:1 (v/v) with a phosphate buffer (75 mM, pH 7.4) and cooled to 279 K in 5 mm NMR tubes prior to measurement. Following daily quality control procedures, including water suppression, quantification, and temperature checking, the samples were analyzed according to Bruker’s in vitro diagnostic research protocol using a 600 MHz Avance III HD NMR spectrometer at 310 K. A 1D nuclear Overhauser effect spectroscopy experiment (pulse program: noesygppr1d) and a 1D Carr-Purcell-Meiboom-Gill spin-echo experiment (pulse program: cpmgpr1d) were acquired for each sample, and 39 metabolites (plus 2 technical additives) as well as 112 lipoproteins were quantified using Bruker’s Quantification in Plasma/Serum (B.I.Quant-PS 2.0.0) and Bruker’s in vitro diagnostic research Lipoprotein Subclass Analysis (B.I.-LISA) software. For lipid profiling, triglycerides, phospholipids, cholesterol, free cholesterol, and apolipoproteins were analyzed in very-low-density lipoprotein (VLDL), intermediate-density lipoprotein (IDL), low-density lipoprotein, and high-density lipoprotein (HDL; Tables S1 and S2 in Multimedia Appendix 1). Although urine samples were collected and analyzed according to standard procedures [21], these findings are not discussed further because the focus of this study is on BG outcomes.

### Data Preprocessing

To facilitate rapid processing by the ML model, 4 essential preprocessing steps were implemented.

#### Data Formatting

In this study, 3 data formats were obtained from the different wearables: AVRO, CSV, and JSON. The CGM device provided IG responses (in mg/dL) in CSV format. EmbracePlus recorded daily physiological data in AVRO and CSV formats, including sensor recordings, time stamps, and frequency rates. To ensure accurate time information during preprocessing, Unix time stamps in GMT were computed and saved with each recording. The recorded value time stamps were calculated using frequency conversion from the initial sensor time stamp. Data collection temporal variations were observed when daily recording start and end times were 15 minutes outside the midnight to 11:59 PM range. Fitbit sensor data were stored in JSON files containing GMT time stamps. Weight, height, age, sex, and BMI were used as demographic variables. The files were merged and converted to NumPy files for data analysis.

#### Data Cleaning

In accordance with standard procedures, the first and last days of CGM data collection were excluded to ensure proper sensor calibration and stabilization. Additionally, variations between participants were observed due to differences in the timing of the initial application and removal of the devices. As a result, our subsequent analyses were conducted using a dataset that comprised 12 of the 14 study days. In consideration of the temporal variability in data collection commencement and conclusion times, we opted to commence our analysis at 1 AM on the second day of the study and conclude it at 11 AM on the 13th day of the study. Afterward, we applied participant-specific outlier detection for artifact removal, that is, values more than 4 SDs from the individual mean were marked as invalid and substituted with the local mean, resulting in the removal of less than 0.2% of data points while preserving physiological validity and temporal continuity.

#### Data Alignment

To properly align the data, missing values, a fluctuating timespan between timesteps, and varying recording frequencies from the sensors had to be compensated for. To address this issue, an artificial timestep array was created. The sampling rate for the features or the prediction horizon (PH) for the labels determined the equidistant timesteps between the fixed start and end times that constrained this array. The primary objective of this approach was to standardize the temporal representation of available data and establish a coherent representation spanning from the start to the end. The next step was to select the data from the sensors that fell within the predefined period. The sensor modality data were then interpolated with a common sampling rate and their respective time information to match the predefined timesteps.

To accommodate the data characteristics of each sensor modality, a customized interpolation approach was adopted. For sensor signals that capture continuous numerical data, such as EDA, taACC, temperature, and plr, linear interpolation (LI) was implemented, with its mathematical formulation provided in equation 1. It estimates intermediate values by constructing a linear polynomial that passes through 2 consecutive known data points, denoted as (x_0_, y_0_) and (x_1_, y_1_). This process yields a piecewise linear representation that approximates the inherent continuous trend of the signal. In this context, $\xi_{LI}(x)$ is the first-order polynomial interpolation operator. Given its modest computational overhead, LI remains a prevalent choice for inferring missing intermediate data points in the dataset.


(1) $$\xi_{LI}(x)=y_{0}\left(\frac{x-x_{1}}{x_{0}-x_{1}}\right)+y_{1}\left(\frac{x-x_{0}}{x_{1}-x_{0}}\right)$$

Notably, LI exhibits discontinuities in smoothness at subinterval boundaries, primarily due to its nondifferentiable nature at these junction points. To address this limitation, we evaluated alternative interpolation techniques for glucose-labeled datasets, where PH was used to define the interval between consecutive data points. One such method tested was nearest neighbor interpolation provided in equation 2. This approach assigns each new data point the value of its nearest existing data point, eliminating the need to compute intermediate values or derivatives. As a result, it retains the step-wise characteristic inherent to the original glucose data.$\text{d}\text{=}\text{arg}{\text{min}}_{\text{i}}\left|\text{x - }{\text{x}}_{\text{i}}\right|$


(2) $$\varepsilon_{NNI}(x)=y_{d},\;d=\arg\min_{i}|x-x_{i}|$$

Here, ${(x}_{i},y_{i})$ is a set of discrete data points for i=0, 1, 2, ... , n. $\xi_{NNI}(x)$ represents the interpolated value at any point x. $arg{min}_{i}\left|x\text{-}x_{i}\right|$ denotes the index d for which the distance $\left|x\text{-}x_{i}\right|$ is the smallest.

#### Data Segmentation

We used a nonoverlapping window segmentation to segment the continuous multivariate time series into samples aligned with the PH, denoted as p. The source data were collected from multiple smartwatch sensors S = [$s_{0}$, $s_{1}$, ... , $s_{n\text{-}1}$] with associated time stamps T = [$t_{0}$, $t_{1}$, ... , $t_{i\text{-}1}$], which were divided into contiguous windows of length p minutes. Each window $k_{x}$ is constructed to encompass the sensor readings from the time interval [$t_{\omega_{x\text{-}p}}$, $t_{\omega_{x}}$] that immediately precedes the corresponding label $\omega_{x}$. This yields a set of windows K = [$k_{0}$, $k_{1}$, ..., $k_{l\text{-}1}$] equal in number to the labels $\Omega \text{=}[\omega_{0},\omega_{1},...,\omega_{l\text{-}1}]$. Consequently, for a fixed observation period, the number of training samples |K| decreases as p increases. This segmentation provides the foundational structure for subsequent supervised learning. All segmentation procedures were executed independently for each participant, ensuring that window construction and temporal indexing remained confined to each individual’s sensor stream and label trajectory.

### Feature Engineering

The complex physiological processes underlying glucose dynamics present a challenge for predicting BG levels [22]. Nevertheless, previous research identified correlations associated with specific extracted features and established the impact of personal demographics on BG responses [23,24]. For each nonoverlapped PH (PH=30 min), handcrafted features were extracted for all sensor modalities, considering their temporal characteristics [25]. In respect thereof, the sensor data were segmented into consecutive 1-minute windows. For every subwindow, a rich set of handcrafted features was computed for each available sensor signal as detailed in Table S3 in Multimedia Appendix 1. These included statistical and time domain descriptors, frequency and amplitude domain measures, and morphological indicators. Temporal context features were added to capture circadian and seasonal regularities. Demographic and anthropometric variables were incorporated to account for interindividual physiological variability. After feature extraction, all variables were concatenated across modalities and time windows to form the final multivariate input representation for the long short-term memory (LSTM) model. In total, 5 contextual and demographic features and 22 features per sensor modality were retained after preprocessing, yielding 159 features per minute per 30-minute observation window for the Empatica dataset and 27 features for the Fitbit dataset. No dimensionality reduction, embedding, or feature selection was applied, as the LSTM architecture was designed to learn hierarchical temporal representations directly from the complete feature space. For more details on the feature engineering standards, see a study by Huang et al [19].

### Model Characteristics

After data preprocessing and feature engineering, we fed the reformulated sensor data (feature vectors) to the LSTM network, which is an enhanced type of recurrent neural network designed to overcome issues such as vanishing gradients and gradient explosions in traditional recurrent neural networks. Using recurrent connections over time, LSTM is capable of capturing and maintaining dynamic temporal information. A key feature of LSTM cells is the use of 3 gating mechanisms, called input gate, forget gate, and output gate, which collectively regulate the flow of information. They use adaptive weights and activation functions to control whether information is retained, updated, or discarded. Specifically, the forget gate uses a sigmoid function to determine whether information from the previous hidden state should be dropped. The input gate, which combines both sigmoid and *tanh* functions, drives which parts of the new input should be stored in the cell state. The output gate then generates the new hidden state based on the updated cell state. In such a manner, the cell state functions as long-term memory, carrying stable information across time steps, while the hidden state serves as short-term memory.

In our implementation, the input to the LSTM consists of 2D sensor data (timesteps×features). The model architecture comprises 4 stacked LSTM layers with progressively decreasing hidden sizes, allowing the network to hierarchically extract temporal representations at different levels of abstraction. After the recurrent layers, the output of the last time step is passed through 2 fully connected layers with a nonlinear *tanh* activation in between, before being projected to the final scalar prediction. This architecture was determined empirically based on preliminary experiments and prior work on glucose forecasting tasks using physiological sensor data [19,26]. Hyperparameters, including the learning rate and hidden layer dimensions, were selected via a grid search performed on the validation partition of each leave-one-subject-out (LOSO) fold to optimize regression performance. This design enables the model to capture complex sequential dependencies while maintaining a compact representation for the final regression output.

### Normalization and Data Leakage Prevention

The prediction task was modeled as a continuous regression problem. To stabilize training and ensure comparability across participants, both the input features and the target glucose values were standardized using fold-specific StandardScaler objects, which were fitted exclusively on the training participants within each LOSO fold. For the target variable, the mean and SD were estimated from the training participants and subsequently applied to the held-out participant. Likewise, each feature dimension was scaled using a StandardScaler fitted on the training data, with the resulting transformation applied to the test participant without incorporating any statistics from the held-out participant. All preprocessing operations that depend on the underlying data distributions, including feature and label standardization, were strictly confined to the training partition of each fold, thereby eliminating any risk of leakage of global statistics into the test data. This procedure ensures that both the features and labels of the held-out participant remain completely independent of the training data, while maintaining stable distributions for LSTM model training and subsequent regression performance evaluation.

### Training Setup

The model was trained for 70 epochs with a batch size of 32 using PyTorch 2.7.1+cu128 in Python 3.11.13 on an NVIDIA RTX 5090 GPU with 32 GB RAM. The optimizer used was Adam (learning rate=0.001), while model optimization was guided by the mean absolute error (MAE; L1 loss), chosen for its stability and robustness to non-Gaussian error distributions in continuous glucose regression. During training, the model output remained in standardized units. Before evaluation, predictions (and targets when required) were transformed back to the original glucose scale using the inverse StandardScaler parameters of the respective LOSO fold to ensure clinically interpretable metrics.

### Quantitative and Qualitative Evaluation

Clinical characteristics were compared between groups using nonparametric Mann-Whitney *U* tests. For BIA measurement and metabolic profiling, a correction for multiple testing using the method of Benjamini et al [27] with a false discovery rate of 1% was performed. Furthermore, linear and multiple linear regression analyses were conducted.

To properly evaluate performance, model effectiveness was examined across different PHs (15, 30, and 60 min) using the proposed LSTM and inverted transformer (iTransformer) models, based on the combined dataset from Empatica and Fitbit wearables. As a benchmark baseline, the iTransformer inverts the architecture by embedding the entire historical time series of each individual variable into an independent token. In detail, the model leverages a multilayer perceptron to transform the temporal sequence of each sensor modality into a high-dimensional vector. The self-attention mechanism is then applied across these variable tokens to capture multivariate correlations, while layer normalization and feed-forward networks characterize the temporal dynamics of each individual variable. This design allows the iTransformer to effectively learn complex intervariable dependencies from heterogeneous multisource wearable data, making it a highly robust benchmark for our study [28]. Algorithm model performance was evaluated using commonly used metrics: MAE, mean absolute percentage error (MAPE), and root-mean-squared error (RMSE). MAE quantifies the average absolute magnitude of errors to reflect overall accuracy, while MAPE measures the percentage deviation to provide a scale-independent metric for relative accuracy. To heavily penalize larger outliers, the mean squared error was computed to assess the average of squared differences. Finally, its square root, RMSE, was reported to express this error in the original measurement units for a more intuitive clinical interpretation. To qualitatively evaluate the model predictions, we performed Clarke error grid analysis (CEGA) [29], in which predefined zone A as clinically accurate predictions that would lead to correct treatment decisions, zone B as deviations that would not result in harmful clinical actions, zones C and D as errors potentially leading to unnecessary overtreatment and failure to treat, respectively, and zone E as misguidance that could cause completely opposite and dangerous interventions.

### CI Calculation

To ensure consistency in statistical comparisons across different cohorts and model conditions, we calculated 95% CIs for all RMSE and MAE metrics. These were derived using a nonparametric bootstrapping method with 1000 resamples. For each cross-validation fold, bootstrap samples were generated by conducting with-replacement random sampling from the model predictions and true glucose values, while maintaining independence at the individual level. In general, SE was calculated as $\text{SD/}\sqrt{\text{n}}$ with n as the number of LOSO folds to reflect the precision of the mean estimate, while 95% CI for RMSE and MAE means were derived from 1000 nonparametric bootstrap resampling, that is, 2.5th and 97.5th percentiles of bootstrap mean distributions. Thereby, narrow CI widths (consistent with SE×2) confirm robust mean performance estimates, even with moderate within-cohort variability. This procedure was consistently applied across all cohorts to ensure statistical comparability.

### Ablation Study of Sensor Modalities

To quantify the individual and combined contribution of each sensor modality to IG prediction performance, a systematic ablation study was implemented. The study was performed on the combined cohort of healthy control (HC) and MR participants using the same preprocessed dataset, LSTM model architecture, training protocol, and LOSO cross-validation strategy as the primary analysis. First, single-modality ablation was conducted by independently removing EDA, PPG, STEMP, and taACC from the feature space. Second, combinatorial ablation tests were performed by removing pairs of sensor modalities to evaluate correlations between modalities. All other features, including demographic variables, temporal context features, and the remaining sensor modalities, were retained without modification. Model performance was evaluated using RMSE, MAE, MAPE, and CEGA to quantify the degradation in predictive accuracy caused by the exclusion of each modality or modality combination, relative to the full-feature model containing all sensor inputs. This approach allowed us to isolate the unique and synergistic predictive value of each physiological signal source for glucose estimation.

### Shapley Additive Explanations Analysis

Shapley Additive Explanations (SHAP) analysis can be used to estimate the marginal importance of input features across all sensor modalities by implementing statistical analysis on the contributions of features, thereby identifying the key physiological mechanisms influencing model predictions. Within the proposed LOSO cross-validation framework, local SHAP values for all features extracted from PPG, EDA, STEMP, and taACC were calculated for each test sample. Following this, the local attribution findings were integrated to generate a global feature importance ranking, demonstrating the impact of each sensor-derived feature on IG prediction. By validating the physiological significance of the hypotheses in the model, we demonstrate the dominant and complementary roles of each modality and ensure that all predictions are based on clinically relevant signal patterns.

## Results

### Study Participant Characteristics

A total of 74 participants participated in our study, including 54 (73%) women and 20 (27%) men. Of these, 34 (46%) individuals were assigned to the HC cohort and 40 (54%) to the MR cohort. The MR cohort comprised individuals who were either overweight and exhibited at least one comorbidity or who were classified as obese. In more detail, individuals with a BMI between 25 and 29.9 kg/m^2^ (overweight) were only included in the MR group if they presented with at least one known metabolic comorbidity, such as hypertension, dyslipidemia, atherosclerosis, insulin resistance, gout, stroke, or heart failure. In contrast, individuals with obesity (BMI ≥30 kg/m^2^) were assigned to the MR cohort regardless of comorbid conditions. As a result, 2 cohorts were formed that exhibited significant differences in their physiological parameters (Table 1).

**Table 1.** Study cohort characteristics^a^.

Characteristics	HC^b^ cohort (n=34)	MR^c^ cohort (n=40)	*P* value
Sex, n (%)			
Female	25 (74)	29 (73)	—^d^
Male	9 (26)	11 (27)	—
Age (years), mean (SD)	24.53 (3.68)	55.38 (15.08)	<.001
BMI (kg/m^2^), mean (SD)	22.36 (2.16)	35.38 (4.91)	<.001
Overweight, n (%)	4 (12)	8 (20)	—
Obesity class 1, n (%)	—	12 (30)	—
Obesity class 2, n (%)	—	14 (35)	—
Obesity class 3, n (%)	—	6 (15)	—
Waist-to-hip ratio, mean (SD)	0.81 (0.08)	0.90 (0.17)	<.001
FM^e^ (kg), mean (SD)	16.03 (4.49)	45.24 (12.31)	<.001
FM (%), mean (SD)	24.16 (5.55)	44.81 (7.61)	<.001
FMI^f^ (kg/m^2^), mean (SD)	5.45 (1.46)	15.93 (4.32)	<.001
SMM^g^ (kg), mean (SD)	23.48 (4.82)	26.47 (6.33)	.045
PA^h^ (°), mean (SD)	5.37 (0.53)	5.00 (0.56)	.01
FFM^i^ (kg), mean (SD)	50.16 (8.67)	54.91 (11.29)	.*08*^j^
FFM (%), mean (SD)	75.84 (5.55)	55.19 (7.61)	<.001
FFMI^k^ (kg/m^2^), mean (SD)	16.93 (1.75)	19.05 (1.68)	<.001
VAT^l^ (L), mean (SD)	0.49 (0.72)	4.21 (2.48)	<.001
WC^m^ (m), mean (SD)	0.73 (0.09)	1.11 (0.13)	<.001
TBW^n^ (L), mean (SD)	36.78 (6.11)	41.28 (8.31)	.01
TBW (%), mean (SD)	55.35 (3.89)	41.21 (5.29)	<.001
ECW^o^ (L), mean (SD)	15.52 (2.37)	18.84 (3.52)	<.001
ECW (%), mean (SD)	23.38 (1.64)	18.83 (1.99)	<.001
ECW/TBW (%), mean (SD)	42.29 (1.67)	45.85 (2.15)	<.001

a Bioelectric impedance analysis (BIA) results were shown for 33 HC and 35 MR individuals, while 1 BIA measurement for HC and 5 measurements for MR were missing. Comparison between metadata and BIA results was performed using multiple Mann-Whitney *U* tests with a false discovery rate (FDR) of 1%.

b HC: healthy control.

c MR: metabolically at-risk.

d Not applicable.

e FM: fat mass.

f FMI: fat mass index.

g SMM: skeletal muscle mass.

h PA: phase angle.

i FFM: fat-free mass.

j Nonsignificance after FDR correction.

k FFMI: fat-free mass index.

l VAT: visceral fat.

m WC: waist circumference.

n TBW: total body water.

o ECW: extracellular water.

The HC cohort comprised 34 individuals, of whom 25 (74%) were women, with a mean age of 24.53 (SD 3.68) years and a BMI within the normal range (mean 22.36, SD 2.16 kg/m²). In contrast, the MR cohort consisted of 40 individuals, including 29 (73%) women, with a mean age of 55.38 (SD 15.08) years and an average BMI in the obese range (mean 35.38, SD 4.91 kg/m²), resulting in significant differences in age and BMI between the 2 cohorts. Within the MR cohort, 8 (20%) participants were overweight with at least one known comorbidity, 12 (30%) presented with obesity class I, 14 (35%) with class II, and 6 (15%) with class III. Both cohorts underwent BIA as well as deep metabolic profiling of blood and urine samples (urine data not shown; Table 1 and Figure 1A). Detailed results of the BIA measurements are presented in Table 1 and Figure 1B. Key parameters linked to metabolic syndrome and disruption in glucose metabolism, including fat-free mass, fat mass, and visceral fat, are highlighted in Figure 1C. Compared to HC, individuals in the MR cohort showed significantly increased fat mass and visceral fat, whereas relative fat-free mass was decreased.

**Figure 1.**
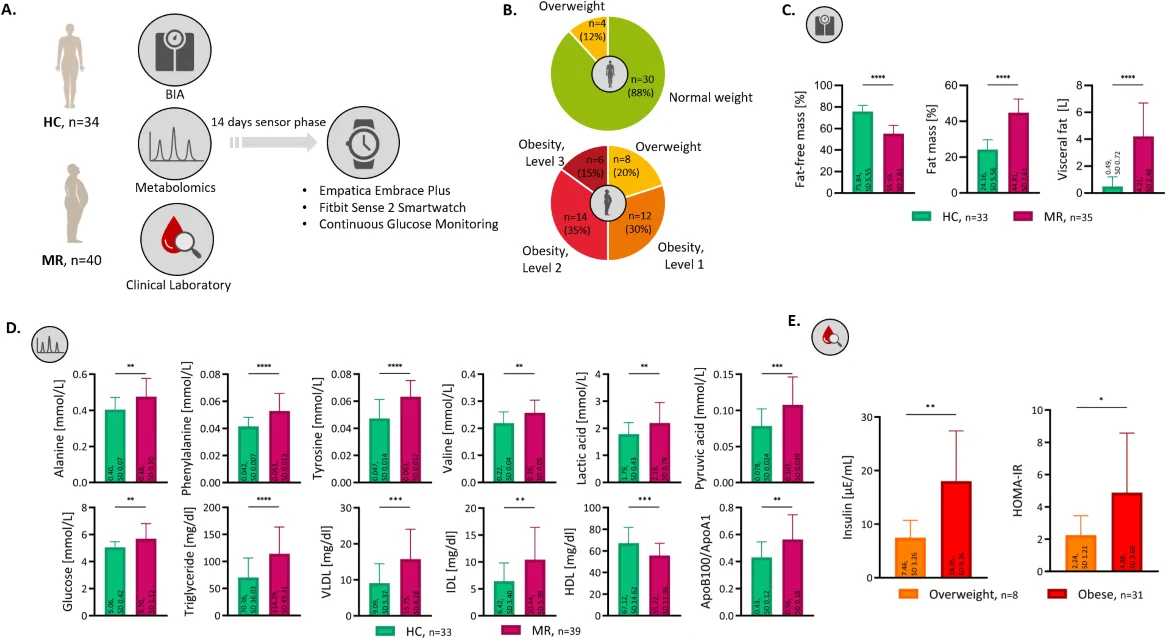
Study design and metabolic markers*.* (A) Study cohort included 74 participants performing a bioelectric impedance analysis (BIA), deep metabolic profiling, and 2-week sensor phase, during which they wore an invasive device for continuous glucose monitoring (CGM) and 2 noninvasive sensor wristbands. (B) Overall cohort was separated into healthy controls (HC) and metabolically at-risk (MR) individuals based on BMI and comorbidities. (C) BIA results indicate alterations in lipid profiles in the MR cohort (n=35) compared to HC (n=33). (D) Blood metabolic phenotyping showed changes in amino acid, glucose, energy, as well as lipid metabolism between MR (n=39) and HC cohorts (n=33). (E) Detailed analysis of glucose metabolism revealed differences among MR individuals with overweight (n=8) and obesity (n=31). Multiple Mann-Whitney *U* test with false discovery rate of 1% for BIA measurement and metabolic profiling as well as 10% for clinical analysis of glucose metabolism were applied with *q≤.1, **q≤.01, ***q≤.001, and ****q<.0001. HDL: high-density lipoprotein; HOMA-IR: homeostatic model assessment of insulin resistance; IDL: intermediate-density lipoprotein; LDL: low-density lipoprotein; VLDL: very-low-density lipoprotein.

Furthermore, deep metabolic profiling of blood samples revealed alterations in amino acid, glucose, energy, as well as lipid metabolism. Elevated levels of amino acids alanine, phenylalanine, tyrosine, and valine were detected in MR compared to HC, along with increased concentrations of lactic and pyruvic acid as well as glucose. In line with the results of the BIA measurement, increased lipid levels (triglycerides, VLDL, and IDL) were observed in MR, whereas HDL was reduced in MR. Furthermore, an increased apolipoprotein ratio (ApoB100/ApoA1) was observed in the MR cohort, serving as a marker for elevated cardiovascular risk (Figure 1D).

To further characterize the overall health status of the MR cohort, additional parameters, including complete blood count, glucose metabolism, liver function, and blood lipids, were assessed by routine clinical laboratory testing (Table 2). Within the MR group, glucose metabolism was analyzed in detail across overweight and obese subgroups, revealing that individuals with obesity exhibited significantly higher insulin concentrations and increased insulin resistance, as indicated by homeostatic model assessment of insulin resistance (Figure 1E). However, no differences were observed between groups for fasting glucose and HbA_1c_.

**Table 2.** Clinical parameters for the metabolically at-risk cohort^a^.

Clinical parameter	Total (n=40)	Overweight (n=8)	Obesity (n=32)
Gender, n (%)			
Women	29 (73)	4 (50)	25 (78)
Men	11 (27)	4 (50)	7 (22)
Large blood count, mean (SD)			
Leukocytes (G/L)	6.66 (1.70)	5.99 (1.10)	6.83 (1.80)
Erythrocytes (T/L)	4.81 (0.35)	5.01 (0.44)	4.76 (0.31)
Hemoglobin (g/dL)	14.34 (1.20)	14.71 (1.22)	14.25 (1.19)
Hematocrit (L/L)	0.42 (0.03)	0.43 (0.03)	0.42 (0.03)
MCH^b^ (pg/Ery)	30.03 (1.90)	29.50 (2.45)	30.16 (1.75)
MCHC^c^ (g/dL)	34.03 (1.22)	34.13 (1.73)	34.00 (1.10)
MCV^d^ (fL)	88.03 (3.87)	86.50 (4.78)	88.42 (3.58)
Thrombocytes (G/L)	267.21 (57.73)	234.25 (30.24)	275.71 (60.37)
Neutrophils (G/L)	3.92 (1.31)	3.71 (1.13)	3.96 (1.35)
Neutrophils (%)	58.14 (8.29)	60.94 (9.21)	57.42 (8.04)
Lymphocytes (G/L)	1.96 (0.70)	1.53 (0.39)	2.08 (0.72)
Lymphocytes (%)	29.75 (7.91)	26.15 (7.51)	30.67 (7.86)
Monocytes (G/L)	0.52 (0.16)	0.54 (0.11)	0.52 (0.17)
Monocytes (%)	7.93 (2.26)	9.04 (1.27)	7.64 (2.38)
Eosinophils (G/L)	0.18 (0.11)	0.17 (0.08)	0.19 (0.12)
Eosinophils (%)	2.91 (1.85)	2.88 (1.41)	2.92 (1.96)
Basophils (G/L)	0.05 (0.03)	0.04 (0.02)	0.05 (0.03)
Basophils (%)	0.84 (0.47)	0.73 (0.54)	0.87 (0.46)
Immature granulocytes (G/L)	0.02 (0.02)	0.02 (0.01)	0.03 (0.02)
Immature granulocytes (%)	0.35 (0.27)	0.28 (0.07)	0.37 (0.30)
Glucose, mean (SD)			
Fasting glucose (mg/dL)	107.56 (19.26)	116.50 (23.16)	105.26 (17.83)
HbA_1c_^e^ (mmol/mol Hb)	38.27 (6.87)	41.43 (10.88)	37.18 (5.07)
HbA_1c_ (%)	5.67 (0.63)	5.98 (0.99)	5.57 (0.46)
Insulin (µE/mL)^f^	15.87 (9.48)	7.46 (3.26)	18.05 (9.36)
HOMA-IR^f^^,^^g^	4.34 (3.49)	2.24 (1.21)	4.88 (3.69)
Liver, mean (SD)			
GOT^h^/ASAT^i^ (U/L)	23.85 (5.89)	22.88 (2.95)	24.10 (6.45)
GPT^j^/ALAT^k^ (U/L)	29.08 (14.06)	20.50 (5.95)	31.29 (14.75)
GGT^l^ (U/L)	30.67 (17.93)	22.88 (13.48)	32.68 (18.56)
Blood lipids, mean (SD)			
Cholesterol (mg/dL)	187.26 (45.37)	203.63 (48.93)	183.03 (44.26)
Triglycerides (mg/dL)	126.92 (50.19)	114.63 (41.05)	130.10 (52.41)
HDL^m^ cholesterol (mg/dL)	53.74 (12.40)	58.38 (12.34)	52.55 (12.33)
Non-HDL cholesterol (mg/dL)	133.51 (41.55)	145.25 (39.67)	130.48 (42.11)
LDL^n^ cholesterol (mg/dL)	108.13 (38.85)	122.38 (41.09)	104.45 (38.07)

a Clinical parameters were shown for 8 participants with overweight and 31 with obesity, while 1 blood sample is missing.

b MCH: mean corpuscular hemoglobin.

c MCHC: mean corpuscular hemoglobin concentration.

d MCV: mean corpuscular volume.

e HbA_1c_: hemoglobin A_1c_.

f Significant difference between participants with overweight and obesity using multiple Mann-Whitney tests with a false discovery rate of 10%.

g HOMA-IR: homeostatic model assessment of insulin resistance.

h GOT: glutamate oxaloacetate transaminase.

i ASAT: aspartate aminotransferase.

j GPT: glutamate pyruvate transaminase.

k ALAT: alanine aminotransferase.

l GGT: gamma glutamyl transferase.

m HDL: high-density lipoprotein.

n LDL: low-density lipoprotein.

### Calculation of IG Values

The aim of the study was to expand our algorithm for the calculation of IG values using noninvasive sensor data and to validate the generalizability of the proposed algorithm under healthy and MR conditions. Therefore, we pursued 2 approaches. First, we implemented 2 cohort-specific algorithms trained and tested for the HC and MR cohorts, respectively. Second, we developed an algorithm using the overall population, consisting of the HC and MR cohorts. For the HC cohort, we achieved a strong performance, indicated by a mean MAE of 13.23 (SD 1.86) mg/dL, a mean MAPE of 0.14 (SD 0.02), a mean RMSE of 17.41 (SD 2.38) mg/dL, and 2.0% of data points in zone D of the CEGA. A total of 33 healthy individuals were included in this dataset, as 1 (3%) participant had to be excluded due to his frequent sauna visits and the resulting errors in data collection. Our MR cohort–specific algorithm also performed well, including 40 individuals, with a mean MAE of 19.75 (SD 8.11) mg/dL, a mean MAPE of 0.18 (SD 0.06), and a mean RMSE of 25.53 (SD 9.28) mg/dL. However, only 1.4% of data points fell in zone D of the CEGA. Furthermore, a cohort-independent algorithm including the HC and MR dataset was developed with a mean MAE of 15.98 (SD 7.19) mg/dL, a mean MAPE of 0.15 (SD 0.05), a mean RMSE of 21.04 (SD 8.32) mg/dL, and 1.7% in zone D of the CEGA (Figure 2A). Model performance characteristics of the overall analysis were stratified by the HC and MR cohorts (Figure 2B), yielding MAE, MAPE, mean squared error, and RMSE values comparable to the cohort-specific analyses. These findings support the assumption that the algorithm is capable of abstracting across the different datasets underscoring its generalizability between various datasets. In addition, we identified a linear association between the RMSE and the HbA_1c_ of the MR cohort (*R*^2^=0.5842; *P*<.001). The simple linear regression analysis remained highly significant (*P*<.001) after the exclusion of the outlier exhibiting an HbA_1c_ value above 7.0%. To further investigate the influence of intersubject variability, we performed a multivariate linear regression analysis with RMSE as the dependent variable and both age and HbA_1c_ as independent variables, indicating that age is not significantly associated with RMSE (β_1_=−0.0146; *P*=.10), while HbA_1c_ shows a strong and statistically significant association with the RMSE (β_2_=12.57; *P*<.001), even after adjusting for age. In line with a previous study by Bent et al [17], we categorized the HbA_1c_ into normal (<5.2%), high normal (5.2%‐5.6%), prediabetic (5.7%‐6.4%), and diabetic (>6.4%), resulting in 82% (n=28 of 33 known values) of individuals with high HbA_1c_ levels and potential disruptions in glucose metabolism (Figure 2C).

**Figure 2.**
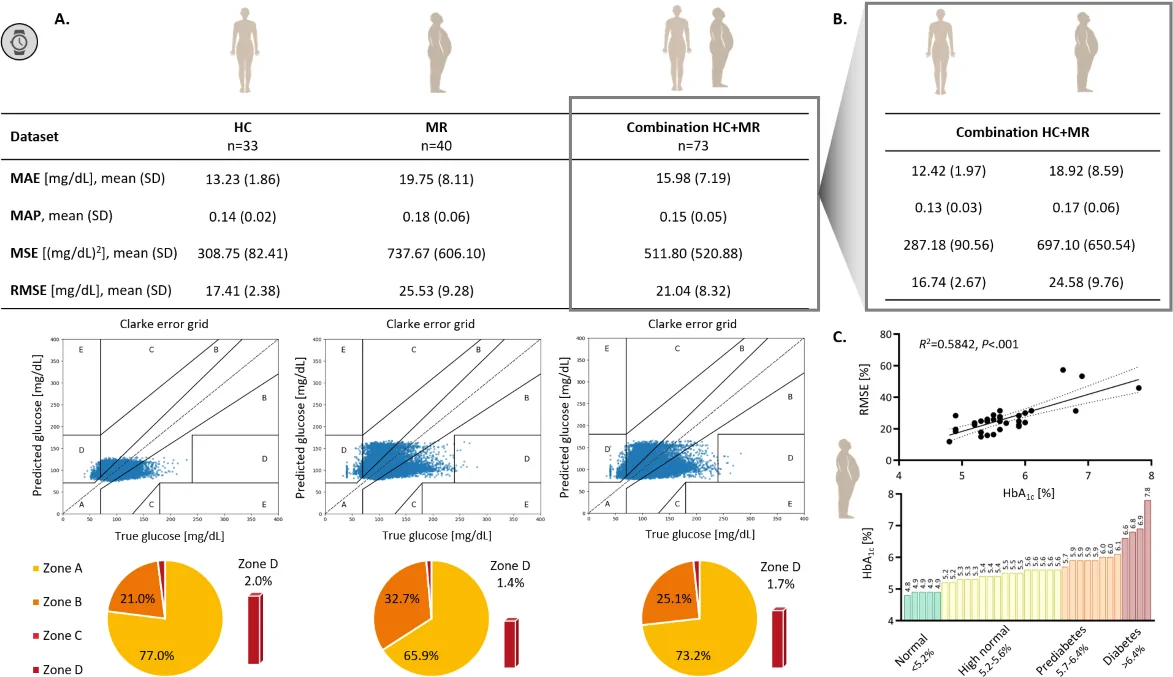
Performance of the algorithm for interstitial blood glucose calculation*.* (A) Model performance was evaluated by mean absolute error (MAE), mean absolute percentage error (MAPE), mean squared error (MSE), root-mean-squared error (RMSE), and Clarke error grid analysis (CEGA) for cohort-specific algorithms of the healthy control (HC) and metabolically at-risk (MR) groups as well as for the combined dataset model. (B) Stratification of the combined model demonstrated its potential for generalizability across different datasets. (C) Linear regression analysis indicated a relationship between the RMSE of the algorithm and abnormalities in blood glucose metabolism, characterized by hemoglobin A_1c_ (HbA_1c_). Furthermore, HbA_1c_ levels of the MR cohort were divided into normal (<5.2%), high normal (5.2%‐5.6%), prediabetic (5.7%‐6.4%), and diabetic (>6.4%).

To evaluate the practical applicability and broad usability of noninvasive IG estimation, the use of a commercially available smartwatch (Fitbit Sense 2) was investigated. Analogous to the evaluation of the sensor data generated by the scientific wristband from Empatica, 3 models were trained: 2 cohort-specific models for the HC and MR groups and 1 combined model. The combined model achieved a mean MAE of 18.91 (SD 10.76) mg/dL, a mean MAPE of 0.18 (SD 0.11), and a mean RMSE of 23.49 (SD 11.46) mg/dL (Figures 3A and 3B). The Fitbit dataset comprised 33 (46%) HC and 38 (54%) participants from the MR cohort, as data from 2 (5% of the overall MR cohort) MR individuals were unavailable due to missing recordings. In line with the Empatica combined model, the combined model based on Fitbit data demonstrated the capability to generalize across different datasets. Furthermore, measured IG values based on CGM as well as the calculated parameters derived from sensor data over a 24-hour period were illustrated for Empatica (Figure 3C) and Fitbit (Figure 3D), showing a high overlap despite visible dynamics in IG values. In summary, the Empatica and Fitbit models exhibited comparable RMSE values, although the commercially available smartwatch showed a slightly reduced performance (Figure 3E).

**Figure 3.**
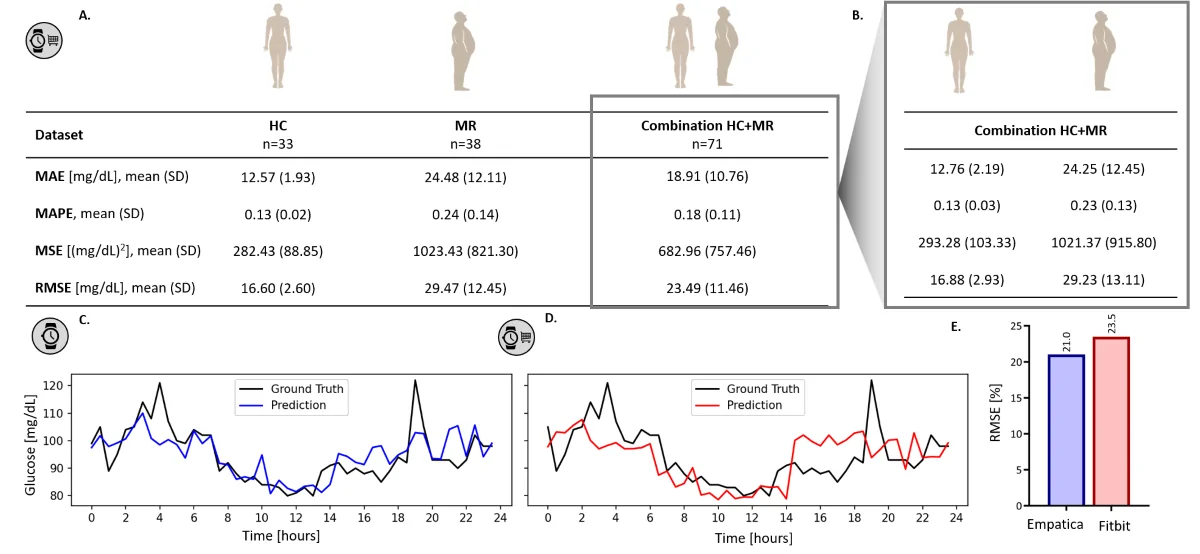
Algorithm performance based on smartwatch data*.* (A) Model performance was evaluated by mean absolute error (MAE), mean absolute percentage error (MAPE), mean squared error (MSE), and root-mean-squared error (RMSE) for the cohort-specific algorithms of the healthy control (HC) and metabolically at-risk (MR) groups as well as characteristics of the combined dataset model. (B) Stratification of the combined model showed the potential for abstraction across different datasets. (C) Interstitial glucose levels measured by a continuous glucose monitoring (CGM) device (black) were compared with values calculated from noninvasive wearable data obtained with the Empatica EmbracePlus (blue) for 1 participant over time. (D) Analogous to C, calculated glucose values were derived from data collected using a commercially available smartwatch (Fitbit Sense 2) (red). (E) Comparison of RMSE between algorithms based on Empatica and Fitbit data for the combined model.

Moreover, to provide additional statistical validation and strengthen comparative claims, SE and 95% CI for both RMSE and MAE were computed using nonparametric bootstrapping with 1000 resamples under the LOSO cross-validation framework for the main cohort analyses, including HC and MR cohorts, as well as the combined cohort, using the proposed LSTM model based on either Empatica or Fitbit data. As shown in Table S4 in Multimedia Appendix 1, the 95% CI for RMSE was 16.34 to 18.48 mg/dL in the HC cohort, 22.65 to 28.41 mg/dL in the MR cohort, 19.12 to 22.96 mg/dL for the combined cohort with Empatica, and 20.81 to 26.17 mg/dL for the combined cohort with Fitbit. Corresponding 95% CIs for MAE were 12.45 to 14.01 mg/dL, 18.12 to 21.38 mg/dL, 14.57 to 17.39 mg/dL, and 17.03 to 20.79 mg/dL, respectively. Despite relatively large SDs across LOSO folds, especially in the MR and combined cohorts, all derived 95% CIs remained narrow, indicating robust and consistent central estimates of model performance. These intervals explicitly quantify statistical uncertainty in overall performance and reinforce that observed between-cohort performance differences are reproducible and not driven by random estimation variability.

To address the influence of PH on IG prediction, we evaluated model performance at 15, 30, and 60 minutes using both Empatica EmbracePlus and Fitbit Sense 2 records (Table S5 in Multimedia Appendix 1). For the Empatica device, the proposed LSTM achieved the lowest RMSE (mean 21.04, SD 8.32 mg/dL) and MAE (mean 15.98, SD 7.19 mg/dL) at a 30-minute PH, with slightly higher errors at 15 minutes (RMSE: mean 21.69, SD 8.30 mg/dL) and 60 minutes (RMSE: mean 22.68, SD 9.01 mg/dL). A similar pattern was observed for the Fitbit device. The 30-minute PH yielded the optimal RMSE (mean 23.49, SD 11.46 mg/dL) compared to 15 minutes (mean 26.10, SD 15.30 mg/dL) and 60 minutes (mean 24.50, SD 11.75 mg/dL). The 30-minute interval was selected as the primary PH because it balances temporal resolution and physiological relevance for PPGR, while minimizing prediction error across both wearable platforms. To validate the LSTM architecture, we further compared it with the iTransformer model at the optimal 30-minute PH (Table S5 in Multimedia Appendix 1). For Empatica data, the LSTM model outperformed iTransformer. For Fitbit, LSTM remained competitive (RMSE: mean 23.49, SD 11.46 vs mean 22.68, SD 8.40 mg/dL) with lower MAE in real-world wearable signals. These results confirm that the LSTM network is better suited for capturing sequential physiological dependencies from continuous wearable data than the transformer-based architecture in this task.

The ablation study confirmed the differential individual and combined contributions of each sensor modality to IG prediction (Table S6 in Multimedia Appendix 1). Single-modality removal showed that PPG and taACC were the most impactful individual features, with their exclusion increasing RMSE by 5.82 and 8.43 mg/dL, respectively, followed by EDA, which led to an RMSE increase of 4.17 mg/dL. STEMP contributed moderately, with an RMSE degradation of 2.35 mg/dL upon elimination. Combinatorial ablation further revealed strong synergistic interactions between modalities. Simultaneous removal of EDA and PPG caused the largest performance drop (RMSE of 9.13 mg/dL), while removing STEMP and taACC resulted in a substantial decline of 7.61 mg/dL, consistent with the high importance of accelerometer-derived motion information. Similarly, excluding PPG and taACC together led to the most severe degradation (RMSE of 10.25 mg/dL), underscoring their dominant and complementary roles. Other combined ablations (EDA+STEMP) also yielded notable accuracy reductions. These findings demonstrate that cardiovascular dynamics from PPG, physical activity patterns from taACC, and autonomic arousal from EDA provide the strongest predictive signals for glucose fluctuations, whereas STEMP acts as a supplementary but meaningful contributor.

To identify the dominant physiological and contextual drivers of glucose prediction, we performed a SHAP analysis on the full dataset (n=39,314), quantifying the magnitude and direction of each feature’s impact on the model’s glucose prediction output, with results visualized in the SHAP beeswarm plot (Figure S1 in Multimedia Appendix 1). It ranks features by the overall magnitude of their SHAP values, revealing that demographic and temporal features were the most influential predictors at the top of the ranking, followed closely by wearable physiological sensor modalities. Focusing on the top 30 most impactful features, we categorized them by sensor modality to quantify their relative contributions, that is, 3 (10%) of the top 30 features were demographic variables, 2 (6.7%) of 30 were temporal metadata, and the remaining 25 (83.3%) of 30 were derived from wearable sensors, encompassing BVP (Plr), EDA, STEMP, and taACC. Within the wearable sensor modalities, accelerometer-derived features were the most abundant, with 12 features (40% of the top 30), followed by 6 (20%) BVP-related features, 4 (13.3%) STEMP features, and 3 (10%) EDA features. The SHAP beeswarm plot further reveals biologically plausible directional effects for these top-ranked features, consistent with well-documented age-related metabolic changes and glucose dysregulation risk. The temporal feature also exhibited a strong directional impact, with high values linked to elevated glucose predictions, reflecting long-term temporal trends in metabolic health and study population dynamics. Wearable physiological features demonstrated consistent dose-response relationships, with high feature values driving predictable shifts in model output, validating their direct physiological relevance to glucose regulation as key complementary functions. In general, these results confirm that demographic and temporal features are the most dominant predictors of glucose levels in our model, with wearable sensor modalities providing critical, physiologically meaningful contributions that enhance predictive accuracy by capturing real-time physiological dynamics.

## Discussion

### Principal Findings

Our study demonstrates the feasibility of predicting IG levels exclusively using noninvasive sensor data and ML techniques. The proposed algorithm achieved high performance across healthy and MR individuals, confirming our previous results [19], even in MR participants with higher glucose levels and dynamics. We implemented cohort-specific algorithms focusing on the HC and MR dataset, respectively, as well as an overall algorithm trained and tested for all individuals together. Furthermore, we identified a potential linear relationship between the error of our algorithms and participants’ glucose metabolism, as characterized by HbA_1c_. This approach may in the future allow for the estimation of HbA_1c_ levels from algorithmic outputs, thereby offering individuals an early indication of possible metabolic dysregulation. In addition, we investigated the potential of using data from commercially available smartwatches for IG prediction. Despite the limited availability of continuous raw data compared with research-grade sensors, we achieved promising results.

Taken together, our IG predictive performance was in line with other state-of-the-art studies, but outperformed these studies in three significant points: (1) to the best of our knowledge, this is the first study using an equal number of healthy and MR individuals resulting in the largest continuous observation with 74 individuals so far; (2) in contrast to other studies, our study exclusively used data collected by wearable smart devices without using manually recorded food logs. Consequently, participants were not required to engage in an additional time-consuming self-reporting, which is prone to data errors; and (3) in addition to sensor wristbands designed for scientific studies, we also tested a commercially available smartwatch in parallel to demonstrate scalability of our proposed solution.

### Comparison to the Literature

Like others, we emphasize the potential of wearable devices for noninvasive glucose prediction. For example, Aziz et al [30] used an open-source dataset consisting of 13 participants aged between 9 and 77 years with a nondiabetic-to-diabetic ratio of 4:6. Various physiological measurements were collected over 3 months, resulting in linear and nonlinear models with high accuracy, specifically an MAE ranging from 0.093 to 0.142 and an RMSE ranging from 0.186 to 0.271. However, the generalizability of this study is limited by the participant-dependent allocation of training and testing as well as the small sample size. Similarly, van den Brink et al [16] conducted a 2-week study with 24 participants without diabetes, using handcrafted features from diet, physical activity, and sleep data to predict glucose levels. They developed a participant-specific Extreme Gradient Boosting model to predict glucose levels with an average MAE of 11.17 mg/dL (reported 0.62, SD 0.15 mmol/L) for the test data. However, this model is also restricted due to the limited generalizability by the personalized training and test strategy. Furthermore, a study by Bent et al [17] involved 16 participants with high normal BG (HbA_1c_=5.2‐5.6) or prediabetes (HbA_1c_=5.7‐6.4) using a CGM sensor, a wristband, and self-reported food logs for 8 to 10 days. They extracted various features and created 2 Extreme Gradient Boosting models—one participant-specific and one population-based—to predict IG levels, achieving an average MAPE of 14.33% (SD 3.25%) and a mean RMSE of 21.22 (SD 4.14) mg/dL for the population model validated with leave-one-person-out cross-validation. Nevertheless, the model is restricted due to the use of self-reported food logs and the limited sample size. Recently, a study published by Zeynali et al [31] demonstrated an RMSE of 19.7 mg/dL using PPG signals of a diverse dataset during surgery and anesthesia. However, although this study included 6388 participants, it comprised only 35,358 BG measurements, corresponding to an average of 5.5 values per participant. In contrast, our longitudinal 2-week dataset comprises 900,606 IG measurements from 74 individuals, providing the potential to capture the temporal dynamics of IG values. Moreover, the glucose data in the study by Zeynali et al were collected during surgical procedures and anesthesia, thus reflecting a highly specific clinical context. A study by Geng et al [32] developed a multisensor-based, noninvasive continuous glucometer with a normalized RMSE of 14.61 mg/dL. However, this study aimed to develop a new sensor device and therefore only 6 healthy individuals and 3 individuals with diabetes were examined for the accuracy of the model. In a study by Zahedani et al [18], an app was developed to identify food types and metabolic factors from images, aiding in glucose prediction. Although this method reduces the burden of self-reporting, it still requires participant involvement, making it unsuitable for long-term monitoring, and its accuracy in complex dietary situations remains unverified. In conclusion, these studies have some limitations in small sample sizes, data processing, and validation and necessitate detailed dietary intake data, requiring significant participant commitment. Without qualitative assessment, the reliability of these ML models for glucose prediction based on clinical validation criteria is challenging.

### Technical Discussion

From the technical point of view, the supplementary 95% CIs derived from nonparametric bootstrapping further validate the stability and generalizability of our prediction model across HC and MR populations. The narrow CIs for RMSE and MAE in all cohorts confirm low between-participant variability and high reproducibility of model performance, rather than reflecting random or spurious estimation. The nonoverlapping tendency of CIs between the HC and MR groups also quantitatively supports the genuine difference in predictive accuracy related to underlying metabolic status, rather than chance variation. Collectively, these statistical estimates strengthen the physiological interpretability of our wearable-based glucose prediction model and reinforce its reliability for real-world mHealth apps in personalized nutrition and metabolic health monitoring.

The optimal performance at a 30-minute PH aligns with the typical time course of postprandial IG excursions, which peak approximately 30 to 60 minutes after meal ingestion. Shorter horizons may lack sufficient physiological signal for robust prediction, while longer horizons (PH=60 min) introduce greater uncertainty due to accumulating variability in lifestyle and metabolic state. Thus, the 30-minute PH provides the best trade-off between predictive accuracy and clinical utility for real-time glycemic monitoring and personalized dietary guidance. Benchmarking against an iTransformer further validates the choice of LSTM for noninvasive IG prediction. While transformers excel at long-range dependencies in static datasets, LSTM networks are more efficient at modeling continuous, noisy physiological time series from wearables, where local temporal dynamics dominate glucose fluctuations. The consistent superiority of LSTM across both research-grade and commercial wearable devices supports its suitability for real-world mHealth apps requiring low latency and robust sequential learning.

Additionally, the findings from ablation tests further strengthen the implied perspectives of the predictive signals underlying noninvasive IG estimation. Individual modality removal revealed that PPG and taACC were the dominant contributors to model accuracy, consistent with the close coupling between cardiovascular tone, physical activity–related metabolic demand, and IG dynamics. EDA also contributed substantially, reflecting the role of autonomic arousal in glucose fluctuation prediction. In contrast, STEMP exerted a more modest effect, supporting its role as a complementary rather than primary predictive feature. Combinatorial ablation confirmed strong synergistic interactions across key modalities. Notably, simultaneous removal of PPG and taACC caused the most severe performance deterioration, while the combined exclusion of EDA and PPG also led to a marked decline, underscoring the value of multimodal integration for robust real-world glucose prediction. These results clarify the relative importance of each wearable signal and validate that our model relies on physiologically meaningful inputs rather than spurious correlations, reinforcing the translational potential of the proposed approach.

### Metabolomics

As a secondary outcome, deep phenotyping of HC and MR individuals was performed. In line with other studies, we identified elevated fat mass and visceral fat in our MR cohort [33,34]. In particular, visceral fat is a well-known biomarker for T2D and cardiovascular risk. Furthermore, blood metabolic profiling revealed alterations in amino acid, energy, and glucose, as well as lipid metabolism. Previous studies showed altered amino acid profiles in T2D compared to HC. For example, a study by Chen et al [35], published in 2019, confirmed our results, highlighting that elevated concentrations of alanine, phenylalanine, tyrosine, and valine increased the risk of T2D. Furthermore, disruptions in glucose metabolism, indicated by increased BG levels, are common biomarkers for the development of insulin resistance, T2D, and metabolic syndrome [36]. Abnormalities in lipid profiles identified by BIA measurement were confirmed by NMR spectroscopy, which indicated increased concentrations of VLDL and IDL, as well as the ratio of ApoA1/ApoA100, a well-known biomarker for cardiovascular risk [37]. In contrast, HDL, commonly referred to as “good cholesterol,” was reduced in MR [38]. Within the MR cohort, individuals with obesity exhibited higher insulin resistance compared to those with overweight, underscoring the association between increased body weight and impaired metabolism [1,2]. This, in turn, may contribute to an elevated risk of insulin resistance, T2D, and metabolic syndrome. Taken together, our deep phenotyping confirms the disruptions in glucose metabolism in the MR cohort and thereby strengthens the validity of our algorithm, which was tested both in healthy individuals and in participants with metabolic impairment.

### Limitations

Nevertheless, further research is needed to validate the IG prediction algorithm. In our study, we examined only a limited cohort consisting of 2 substudies with different study populations, including differences in demographic characteristics such as age and BMI. Furthermore, we were able to test only one commercial smartwatch and did not cover other wearables and smartwatches on the market. Therefore, it is appropriate to evaluate the efficacy in larger and more diverse cohorts, as well as testing the model on distinct sensor data collected from various smart devices is essential for detailed fine-tuning to reduce the impact of data diversity on IG prediction outcomes. This includes validating the algorithm in different clinical scenarios following the V3 [39] principle and adapting it for predicting IG response in metabolic diseases such as obesity and T2D. Furthermore, the role of confounding factors such as age, gender, BMI, dietary intake, physical activity, stress, sleep, and medication should be evaluated. In particular, the observed relationship between RMSE and HbA_1c_ levels, as well as a possible association with other confounders, warrants further consideration. On the one hand, this observation may indicate reduced model performance in persons with strong fluctuations in IG levels, while on the other hand, it may suggest a potential for noninvasive estimation of HbA_1c_ levels, which could be of interest for prevention and early detection of abnormalities in glucose metabolism. It is also critical to rigorously validate the extracted features to ensure that predictive signals reflect true physiological responses rather than artifacts or spurious correlations. In particular, when using smartwatch data with limited output parameters, it is important to verify that physiological signals actually reflect the IG values. A limitation of this study is that the model was evaluated primarily in terms of pointwise glucose prediction accuracy. Dynamic characteristics such as trend direction, rate of change, and peak timing were not explicitly assessed. These aspects are important for clinical decision-making and should be investigated in future work. From the technical point, exploring AI learning strategies with promising potential in processing physiological signals, including updated Neural Hierarchical Interpolation for Time Series [40], transfer learning [41], or federated learning [42], is warranted. Moreover, combining multimodal data sources such as continuous activity, sleep, and heart rate metrics could further enhance feature validity and predictive accuracy across diverse populations. In the long term, systematic assessment of the algorithm’s sensitivity to lifestyle and dietary variations is necessary to ensure its robustness for real-world mHealth apps and PLGD recommendations.

### Conclusions

In conclusion, we predicted continuous IG values using wearable technologies and ML approaches, resulting in an algorithm obtaining strong performance with an average MAE of 15.98 (SD 7.19) mg/dL, a mean MAPE of 0.15 (SD 0.05), a mean RMSE of 21.04 (SD 8.32) mg/dL, and 1.7% of predictive points in zone D of the CEGA. Notably, this performance was achieved using one of the largest datasets consisting of HC and MR individuals without relying on food logs, which sets our study apart from others. The widespread availability of wearables and smartwatches offers a scalable and cost-efficient path toward broad CGM-based monitoring, supporting PLGD strategies and enabling early risk detection. Leveraging longitudinal multimodal data may further enhance predictive accuracy and extend the applicability of PLGD approaches to other conditions associated with metabolic dysregulation, including neurodegenerative diseases.

## Supplementary material

10.2196/91724 Multimedia Appendix 1 Additional tables with supplementary results.

## Abbreviations

BG: blood glucose
BIA: bioelectrical impedance analysis
BVP: blood volume pulse
CEGA: Clarke error grid analysis
CGM: continuous glucose monitoring
EDA: electrodermal activity
HbA1c: hemoglobin A1c
HC: healthy control
HDL: high-density lipoprotein
IDL: intermediate-density lipoprotein
IG: interstitial glucose
iTransformer: inverted transformer
LI: linear interpolation
LOSO: leave-one-subject-out
LSTM: long short-term memory
MAE: mean absolute error
MAPE: mean absolute percentage error
mHealth: mobile health
ML: machine learning
MR: metabolically at-risk
NMR: nuclear magnetic resonance
PH: prediction horizon
PLGD: postprandial low-glycemic diet
Plr: pulse rate
PPG: photoplethysmography
PPGR: postprandial glucose response
RMSE: root-mean-squared error
SHAP: Shapley Additive Explanations
STEMP: skin temperature
T2D: type 2 diabetes
taACC: triaxial acceleration
VLDL: very-low-density lipoprotein
