# Supplementary material for: Noninvasive Interstitial Glucose Estimation Using Wearables and Machine Learning in Healthy Individuals and Individuals With Obesity: Observational Cohort Study
Source: JMIR Mhealth Uhealth. 2026 Sep 30;14:e91724. doi: 10.2196/91724 (PMC13626402; doi:10.2196/91724)
Supplement: Supplementary file 1 [file mhealth-v14-e91724-s001.pdf]

Non-invasive Interstitial Glucose Estimation using Wearables and Machine Learning in Healthy and Obese Individuals: A Proof-of-Concept Study

Franziska Schmelter, Xinyu Huang, Annika Heidmann, Paul Beier, Friederike Miriam Rundfeldt, Christina Plöger, Artur Piet, Md Abid Hasan, Yuanheng Zhang, Lennart Jablonski, Oliver Witt, Torsten Schröder, Stefanie Derer, Chris Easthope Awai, Marcin Grzegorzek and Christian Sina

Supplementary Table S1: Metabolites in blood, their limit of detection (LOD), and typical concentration ranges (95% range) that are standardly quantified by Bruker's *in-vitro* diagnostic research (IVDr) procedure and comparison of the blood metabolome profiles from healthy controls (HC) and metabolically at-risk (MR) individuals using unpaired, non-parametric Mann-Whitney test with a false discovery rate (FDR) of 1% for multiple comparisons.

Parameter	Limit of Detection (LOD) [mmol/L]	95% Range [mmol/L]	HC *vs.* MR *q*-value
**Alcohols and Derivatives**			
Ethanol	0.1	≤ 0.82	0.600719
**Amines and Derivatives**			
Trimethylamine-N-oxide	0.08	≤ 0.08	0.351045
**Amino acids and Derivatives**			
2-Aminobutyric acid	0.05	≤ 0.10	0.565376
Alanine	0.02	0.29 - 0.64	0.001913
Asparagine	0.05	≤ 0.08	0.002680
Creatine	0.01	≤ 0.07	0.000732
Creatinine	0.01	0.06 - 0.14	0.000008
Glutamic acid	0.05	≤ 0.24	0.000023
Glutamine	0.02	0.30 - 0.83	0.027672
Glycine	0.01	0.17 - 0.44	0.477057
Histidine	0.02	0.07 - 0.16	0.051206
Isoleucine	0.03	0.03 - 0.11	0.168372
Leucine	0.01	0.07 - 0.20	0.022509
Lysine	0.04	≤ 0.29	0.019391
Methionine	0.05	0.05 - 0.13	0.352858
N,N-Dimethylglycine	0.01	≤ 0.01	0.600719
Ornithine	0.02	≤ 0.16	0.221858
Phenylalanine	0.03	≤ 0.07	0.000092
Proline	0.05	≤ 0.59	0.153740
Sarcosine	0.01	≤ 0.01	0.600719
Threonine	0.04	≤ 0.24,	0.498722
Tyrosine	0.03	≤ 0.08	0.000008
Valine	0.03	0.15 - 0.35	0.002070
**Carboxylic acids**			
2-Hydroxybutyric acid	0.15	≤ 0.17	0.354862
Acetic acid	0.01	≤ 0.06	0.000144
Citric acid	0.03	≤ 0.21	0.201628
Formic acid	0.02	≤ 0.03	0.354862
Lactic acid	0.03	2.23 - 7.14	0.004319
Succinic acid	0.01	≤ 0.01	0.600719

Parameter	Limit of Detection (LOD) [mmol/L]	95% Range [mmol/L]	HC *vs.* MR *q*-value
**Essential nutrient**			
Choline	0.05	≤ 0.06	0.600719
**Keto acids and Derivatives**			
2-Oxoglutaric acid	0.02	≤ 0.02	0.600719
3-Hydroxybutyric acid	0.02	≤ 0.26	0.361301
Acetoacetic acid	0.01	≤ 0.02	0.130925
Acetone	0.01	≤ 0.06	0.025390
Pyruvic acid	0.03	≤ 0.07	0.000436
**Sugar and Derivatives**			
D-Galactose	0.11	≤ 0.11	0.168372
Glucose	0.54	1.73 - 6.08	0.003156
Glycerol	0.08	≤ 0.44	0.004906
**Sulfones**			
Dimethylsulfone	0.01	≤ 0.02	0.026450
**Technical Additives**			
Ca-EDTA	0.5	1.90 - 2.60	-
K-EDTA	0.5	4.50 - 7.50	-

Supplementary Table S2: Lipoproteins in blood, their common clinical name, abbreviation, and typical concentration ranges (95% range) that are standardly quantified by Bruker's *in-vitro* diagnostic research (IVDr) procedure and comparison of the blood lipid profiles from healthy controls (HC) and metabolically at-risk (MR) individuals using unpaired, non-parametric Mann-Whitney test with a false discovery rate (FDR) of 1% for multiple comparisons.

Key	Parameter	Clinical Name	Abbreviation	95% Range	HC *vs.* MR *q*-value
**Main Parameters [mg/dL]**					
TPTG	TG	Triglycerides	TG	53.45 - 489.81	0.000063
TPCH	Chol	Total Cholesterol	Chol	140.31 - 341.43	0.517639
LDCH	LDL-Chol	LDL Cholesterol	LDL	54.52 - 226.60	0.498557
HDCH	HDL-Chol	HDL Cholesterol	HDL	34.60 - 96.25	0.000163
TPA1	Apo-A1	Apolipoprotein A1	ApoA1	112.00 - 216.85	0.016125
TPA2	Apo-A2	Apolipoprotein A2	ApoA2	24.07 - 47.70	0.037212
TPAB	Apo-B100	Apolipoprotein B100	ApoB100	48.18 - 159.94	0.017726
**Calculated Parameters [nmol/L]**					
LDHD	LDL-Chol/HDL-Chol	LDL/HDL ratio	LDL/HDL	0.98 - 4.08	0.037212
ABA1	Apo-B100/Apo-A1	ApoB100/ApoA1 ratio	ApoB100/ApoA1	0.30 - 1.07	0.002070
TBPN	Total Particle Number	Total ApoB100 Carrying Particles	ApoB Particles	876.01 - 2908.20	0.017726
VLPN	VLDL Particle Number	VLDL Particle Number	VLDL Particles	50.11 - 473.04	0.000063
IDPN	IDL Particle Number	IDL Particle Number	IDL Particles	35.95 - 316.39	0.000175

Key	Parameter	Clinical Name	Abbreviation	95% Range	HC *vs.* MR *q*-value
LDPN	LDL Particle Number	LDL Particle Number	LDL Particles	760.42 - 2559.51	0.116146
L1PN	LDL-1 Particle Number	LDL-1 Particle Number	LDL-1 Particles	98.10 - 567.23	0.121717
L2PN	LDL-2 Particle Number	LDL-2 Particle Number	LDL-2 Particles	46.65 - 426.74	0.067113
L3PN	LDL-3 Particle Number	LDL-3 Particle Number	LDL-3 Particles	51.24 - 499.00	0.425091
L4PN	LDL-4 Particle Number	LDL-4 Particle Number	LDL-4 Particles	77.07 - 577.04	0.044969
L5PN	LDL-5 Particle Number	LDL-5 Particle Number	LDL-5 Particles	85.60 - 614.86	0.016881
L6PN	LDL-6 Particle Number	LDL-6 Particle Number	LDL-6 Particles	90.64 - 815.44	0.390567
**Lipoprotein Main Fractions [mg/dL]**					
VLTG	VLDL	VLDL Triglycerides	VLDL TG	21.38 - 335.60	0.000144
IDTG	IDL	IDL Triglycerides	IDL TG	4.62 - 100.01	0.000094
LDTG	LDL	LDL Triglycerides	LDL TG	11.77 - 45.22	0.000165
HDTG	HDL	HDL Triglycerides	HDL TG	7.29 - 28.58	0.000819
VLCH	VLDL	VLDL Cholesterol	VLDL	4.88 - 76.99	0.000397
IDCH	IDL	IDL Cholesterol	IDL	3.91 - 49.96	0.002070
LDCH	LDL	LDL Cholesterol	LDL	54.52 - 226.60	0.498557
HDCH	HDL	HDL Cholesterol	HDL	34.60 - 96.25	0.000163
VLFC	VLDL	VLDL Free Cholesterol	VLDL FC	2.66 - 33.09	0.000165
IDFC	IDL	IDL Free Cholesterol	IDL FC	0.94 - 13.89	0.001759
LDFC	LDL	LDL Free Cholesterol	LDL FC	17.19 - 63.35	0.498557
HDFC	HDL	HDL Free Cholesterol	HDL FC	6.98 - 27.05	0.000160
VLPL	VLDL	VLDL Phospholipids	VLDL PL	6.44 - 67.58	0.000094
IDPL	IDL	IDL Phospholipids	IDL PL	2.97 - 32.53	0.004906
LDPL	LDL	LDL Phospholipids	LDL PL	36.69 - 120.76	0.398256
HDPL	HDL	HDL Phospholipids	HDL PL	56.50 - 135.93	0.017726
HDA1	HDL	HDL Apolipoprotein A1	HDL ApoA1	110.04 - 222.02	0.007031
HDA2	HDL	HDL Apolipoprotein A2	HDL ApoA2	24.86 - 47.99	0.110000
VLAB	VLDL	VLDL Apolipoprotein B	VLDL ApoB	2.76 - 26.02	0.000063
IDAB	IDL	IDL Apolipoprotein B	IDL ApoB	1.98 - 17.40	0.000175
LDAB	LDL	LDL Apolipoprotein B	LDL ApoB	41.82 - 140.77	0.116146
**VLDL Subfractions [mg/dL]**					
V1TG	VLDL-1	VLDL-1 Triglycerides	VLDL-1 TG	6.23 - 212.19	0.000135

Key	Parameter	Clinical Name	Abbreviation	95% Range	HC *vs.* MR *q*-value
V2TG	VLDL-2	VLDL-2 Triglycerides	VLDL-2 TG	2.75 - 66.92	0.000732
V3TG	VLDL-3	VLDL-3 Triglycerides	VLDL-3 TG	2.16 - 48.88	0.004753
V4TG	VLDL-4	VLDL-4 Triglycerides	VLDL-4 TG	2.93 - 28.43	0.000337
V5TG	VLDL-5	VLDL-5 Triglycerides	VLDL-5 TG	1.08 - 7.26	0.000148
V1CH	VLDL-1	VLDL-1 Cholesterol	VLDL-1 Chol	0.80 - 35.13	0.000337
V2CH	VLDL-2	VLDL-2 Cholesterol	VLDL-2 Chol	0.39 - 15.36	0.038486
V3CH	VLDL-3	VLDL-3 Cholesterol	VLDL-3 Chol	0.48 - 16.23	0.014921
V4CH	VLDL-4	VLDL-4 Cholesterol	VLDL-4 Chol	1.41 - 15.11	0.000566
V5CH	VLDL-5	VLDL-5 Cholesterol	VLDL-5 Chol	0.10 - 3.93	0.000067
V1FC	VLDL-1	VLDL-1 Free Cholesterol	VLDL-1 FC	0.11 - 12.89	0.000092
V2FC	VLDL-2	VLDL-2 Free Cholesterol	VLDL-2 FC	0.04 - 6.60	0.011069
V3FC	VLDL-3	VLDL-3 Free Cholesterol	VLDL-3 FC	0.05 - 8.09	0.004753
V4FC	VLDL-4	VLDL-4 Free Cholesterol	VLDL-4 FC	0.15 - 7.21	0.004906
V5FC	VLDL-5	VLDL-5 Free Cholesterol	VLDL-5 FC	0.02 - 2.20	0.003811
V1PL	VLDL-1	VLDL-1 Phospholipids	VLDL-1 PL	1.31 - 32.41	0.000094
V2PL	VLDL-2	VLDL-2 Phospholipids	VLDL-2 PL	0.81 - 15.43	0.000819
V3PL	VLDL-3	VLDL-3 Phospholipids	VLDL-3 PL	0.82 - 13.53	0.001913
V4PL	VLDL-4	VLDL-4 Phospholipids	VLDL-4 PL	1.62 - 12.87	0.000326
V5PL	VLDL-5	VLDL-5 Phospholipids	VLDL-5 PL	0.40 - 5.06	0.000023
**LDL Subfractions [mg/dL]**					
L1TG	LDL-1	LDL-1 Triglycerides	LDL-1 TG	2.51 - 14.03	0.000023
L2TG	LDL-2	LDL-2 Triglycerides	LDL-2 TG	1.19 - 6.40	0.034932
L3TG	LDL-3	LDL-3 Triglycerides	LDL-3 TG	1.15 - 5.72	0.012354
L4TG	LDL-4	LDL-4 Triglycerides	LDL-4 TG	1.21 - 8.43	0.001235
L5TG	LDL-5	LDL-5 Triglycerides	LDL-5 TG	1.12 - 8.98	0.001030
L6TG	LDL-6	LDL-6 Triglycerides	LDL-6 TG	1.35 - 13.16	0.103610
L1CH	LDL-1	LDL-1 Cholesterol	LDL-1 Chol	8.07 - 58.72	0.387282
L2CH	LDL-2	LDL-2 Cholesterol	LDL-2 Chol	2.48 - 48.09	0.022509
L3CH	LDL-3	LDL-3 Cholesterol	LDL-3 Chol	3.19 - 45.64	0.161938
L4CH	LDL-4	LDL-4 Cholesterol	LDL-4 Chol	4.32 - 48.86	0.126621
L5CH	LDL-5	LDL-5 Cholesterol	LDL-5 Chol	5.41 - 48.69	0.037585

Key	Parameter	Clinical Name	Abbreviation	95% Range	HC *vs.* MR *q*-value
L6CH	LDL-6	LDL-6 Cholesterol	LDL-6 Chol	6.26 - 54.19	0.367665
L1FC	LDL-1	LDL-1 Free Cholesterol	LDL-1 FC	2.49 - 16.94	0.517639
L2FC	LDL-2	LDL-2 Free Cholesterol	LDL-2 FC	0.99 - 14.35	0.002583
L3FC	LDL-3	LDL-3 Free Cholesterol	LDL-3 FC	1.27 - 13.29	0.028789
L4FC	LDL-4	LDL-4 Free Cholesterol	LDL-4 FC	1.07 - 12.49	0.404205
L5FC	LDL-5	LDL-5 Free Cholesterol	LDL-5 FC	1.56 - 12.71	0.132512
L6FC	LDL-6	LDL-6 Free Cholesterol	LDL-6 FC	1.78 - 12.18	0.515099
L1PL	LDL-1	LDL-1 Phospholipids	LDL-1 PL	5.87 - 29.80	0.165515
L2PL	LDL-2	LDL-2 Phospholipids	LDL-2 PL	2.20 - 24.82	0.026949
L3PL	LDL-3	LDL-3 Phospholipids	LDL-3 PL	2.39 - 24.21	0.251893
L4PL	LDL-4	LDL-4 Phospholipids	LDL-4 PL	3.05 - 25.06	0.068541
L5PL	LDL-5	LDL-5 Phospholipids	LDL-5 PL	3.72 - 25.29	0.026005
L6PL	LDL-6	LDL-6 Phospholipids	LDL-6 PL	4.44 - 27.91	0.401918
L1AB	LDL-1	LDL-1 Apolipoprotein B	LDL-1 ApoB	5.40 - 31.20	0.120894
L2AB	LDL-2	LDL-2 Apolipoprotein B	LDL-2 ApoB	2.57 - 23.47	0.065704
L3AB	LDL-3	LDL-3 Apolipoprotein B	LDL-3 ApoB	2.82 - 27.44	0.425091
L4AB	LDL-4	LDL-4 Apolipoprotein B	LDL-4 ApoB	4.24 - 31.74	0.045122
L5AB	LDL-5	LDL-5 Apolipoprotein B	LDL-5 ApoB	4.71 - 33.82	0.016881
L6AB	LDL-6	LDL-6 Apolipoprotein B	LDL-6 ApoB	4.98 - 44.85	0.390567
**HDL Subfractions [mg/dL]**					
H1TG	HDL-1	HDL-1 Triglycerides	HDL-1 TG	1.40 - 11.96	0.179510
H2TG	HDL-2	HDL-2 Triglycerides	HDL-2 TG	0.98 - 5.47	0.001952
H3TG	HDL-3	HDL-3 Triglycerides	HDL-3 TG	1.30 - 5.49	0.000397
H4TG	HDL-4	HDL-4 Triglycerides	HDL-4 TG	1.94 - 8.49	0.000516
H1CH	HDL-1	HDL-1 Cholesterol	HDL-1 Chol	6.10 - 46.07	0.004906
H2CH	HDL-2	HDL-2 Cholesterol	HDL-2 Chol	3.98 - 15.58	0.008014
H3CH	HDL-3	HDL-3 Cholesterol	HDL-3 Chol	6.82 - 18.79	0.010710
H4CH	HDL-4	HDL-4 Cholesterol	HDL-4 Chol	10.64 - 30.26	0.302207
H1FC	HDL-1	HDL-1 Free Cholesterol	HDL-1 FC	1.45 - 11.96	0.000063
H2FC	HDL-2	HDL-2 Free Cholesterol	HDL-2 FC	0.74 - 4.59	0.000063
H3FC	HDL-3	HDL-3 Free Cholesterol	HDL-3 FC	1.25 - 5.27	0.004121

Key	Parameter	Clinical Name	Abbreviation	95% Range	HC *vs.* MR *q*-value
H4FC	HDL-4	HDL-4 Free Cholesterol	HDL-4 FC	2.13 - 8.54	0.152029
H1PL	HDL-1	HDL-1 Phospholipids	HDL-1 PL	7.67 - 57.17	0.008715
H2PL	HDL-2	HDL-2 Phospholipids	HDL-2 PL	7.40 - 26.82	0.116146
H3PL	HDL-3	HDL-3 Phospholipids	HDL-3 PL	12.03 - 31.53	0.408420
H4PL	HDL-4	HDL-4 Phospholipids	HDL-4 PL	19.75 - 43.53	0.408420
H1A1	HDL-1	HDL-1 Apolipoprotein A1	HDL-1 ApoA1	5.95 - 75.40	0.002547
H2A1	HDL-2	HDL-2 Apolipoprotein A1	HDL-2 ApoA1	9.94 - 36.22	0.109868
H3A1	HDL-3	HDL-3 Apolipoprotein A1	HDL-3 ApoA1	18.26 - 47.09	0.408420
H4A1	HDL-4	HDL-4 Apolipoprotein A1	HDL-4 ApoA1	56.03 - 110.49	0.449121
H1A2	HDL-1	HDL-1 Apolipoprotein A2	HDL-1 ApoA2	0.77 - 8.31	0.004906
H2A2	HDL-2	HDL-2 Apolipoprotein A2	HDL-2 ApoA2	1.88 - 7.78	0.165347
H3A2	HDL-3	HDL-3 Apolipoprotein A2	HDL-3 ApoA2	4.85 - 11.84	0.314242
H4A2	HDL-4	HDL-4 Apolipoprotein A2	HDL-4 ApoA2	12.02 - 29.58	0.497994

Abbreviations: Apo = Apolipoproteins; Chol = Cholesterol; FC = Free Cholesterol; HDL = High-Density Lipoprotein; IDL = Intermediate-Density Lipoprotein; LDL= Low-Density Lipoprotein; PL = phospholipids; TG = Triglycerides; VLDL = Very Low-Density Lipoprotein.

Supplementary Table S3: Overview of features extracted per one-minute segment within a 30-minute observation window.

Feature	Category	Description	Notes
minutes_from_midnight	Temporal Context	Number of minutes from midnight of the current day	
days_from_beginning_of_year	Temporal Context	Number of days since January 1	
demographic_age	Demographic / Anthropometric	Age in years	
demographic_sex	Demographic / Anthropometric	Biological sex, 0 = male, 1 = female	
demographic_bmi	Demographic / Anthropometric	Body mass index, based on height and weight	
mean	Statistical / Time-domain	Mean of the sensor signal	Computed per sensor modality
max	Statistical / Time-domain	Maximum value of the sensor signal	Computed per sensor modality
argmax	Statistical / Time-domain	Index of the maximum value	Computed per sensor modality

Feature	Category	Description	Notes
min	Statistical / Time-domain	Minimum value of the sensor signal	Computed per sensor modality
argmin	Statistical / Time-domain	Index of the minimum value	Computed per sensor modality
std	Statistical / Time-domain	Standard deviation of the signal	Computed per sensor modality
median	Statistical / Time-domain	Median of the signal	Computed per sensor modality
zero_crossings	Statistical / Time-domain	Number of times the signal crosses zero	Computed per sensor modality
q20	Statistical / Time-domain	20th percentile of the signal	Computed per sensor modality
q50	Statistical / Time-domain	50th percentile of the signal	Computed per sensor modality
q80	Statistical / Time-domain	80th percentile of the signal	Computed per sensor modality
iqr	Statistical / Time-domain	Interquartile range (75th - 25th percentile)	Computed per sensor modality
autocorr	Statistical / Time-domain	Lag-1 autocorrelation of the signal	Computed per sensor modality
kurtosis	Statistical / Time-domain	Measure of peakedness of the signal	Computed per sensor modality
entropy	Statistical / Time-domain	Shannon entropy of the signal	Computed per sensor modality
fft_mean	Frequency / Amplitude-domain	Mean of absolute FFT coefficients	Computed per sensor modality
fft_std	Frequency / Amplitude-domain	Standard deviation of absolute FFT coefficients	Computed per sensor modality
spectral_energy	Frequency / Amplitude-domain	Total signal energy estimated via Welch PSD	Computed per sensor modality
spectral_entropy	Frequency / Amplitude-domain	Spectral entropy of the signal via Welch PSD	Computed per sensor modality
wavelet_energy	Frequency / Amplitude-domain	Sum of squared wavelet coefficients (db1)	Computed per sensor modality
peak_count	Morphological	Number of peaks in the signal	Computed per sensor modality
pit_count	Morphological	Number of valleys in the signal	Computed per sensor modality

Supplementary Table S4: Confidence interval (CI) calculations using the proposed long short-term memory (LSTM) model based on different wearables and datasets, evaluated by root mean square error (RMSE) and mean absolute error (MAE) of the 95% CI for healthy controls (HC) and metabolically at-risk (MR) participants.

**Dataset**	**RMSE (mg/dL)**	**RMSE - SE (mg/dL)**	**95% CI for RMSE**	**MAE (mg/dL)**	**MAE - SE (mg/dL)**	**95% CI for MAE**
HC cohort (Empatica)	17.41 ± 2.38	0.58	[16.34, 18.48]	13.23 ± 1.86	0.45	[12.45, 14.01]
MR cohort (Empatica)	25.53 ± 9.28	1.44	[22.65, 28.41]	19.75 ± 8.11	1.26	[18.12, 21.38]
Combined cohort (Empatica)	21.04 ± 8.32	0.97	[19.12, 22.96]	15.98 ± 7.19	0.87	[14.57, 17.39]
Combined cohort (Fitbit)	23.49 ± 11.46	1.18	[20.81, 26.17]	18.91 ± 10.76	1.31	[17.03, 20.79]

**Note: Standard error (SE) was calculated as* $\mathrm{SD}/\sqrt{\mathrm{n}}$ *(n = number of LOSO folds) to reflect the precision of the mean estimate, while 95% CI for RMSE and MAE means were derived from 1,000 non-parametric bootstrap resamplings, i.e., 2.5th and 97.5th percentiles of bootstrap mean distributions. The narrow CI widths (consistent with SE×2) confirm robust mean performance estimates, even with moderate within-cohort variability (SD).*

Supplementary Table S5: Model performance across different prediction horizons (PH) using proposed long short-term memory (LSTM) and inverted transformer (iTransformer models) based on combined dataset with Empatica and Fitbit wearables. Optimal performance at **30 min PH** is highlighted in bold.

**Device**	**Model**	**PH**	**MAE (mg/dL)**	**MAPE**	**RMSE (mg/dL)**
Empatica Embrace Plus	LSTM	15 min	16.38 ± 6.86	0.16 ± 0.05	21.69 ± 8.30
	**<u>LSTM</u>**	**30 min**	**15.98 ± 7.19**	**0.15 ± 0.05**	**21.04 ± 8.32**
	LSTM	60 min	17.14 ± 7.58	0.16 ± 0.06	22.68 ± 9.01
	iTransformer	30 min	17.33 ± 6.18	0.17 ± 0.04	22.38 ± 7.35
Fitbit Sense 2	LSTM	15 min	21.07 ± 14.79	0.21 ± 0.15	26.10 ± 15.30
	LSTM	30 min	18.91 ± 10.76	0.18 ± 0.11	23.49 ± 11.46
	LSTM	60 min	19.61 ± 11.21	0.19 ± 0.11	24.50 ± 11.75
	iTransformer	30 min	17.59 ± 7.31	0.17 ± 0.05	22.68 ± 8.40

Supplementary Table S6: Ablation study performance comparison using proposed long short-term memory (LSTM) model with 30-minute prediction horizon (PH) between different senor modalities on the combined (HC+MR) dataset.

**Modality combination**	**RMSE (mg/dL)**	**MAE (mg/dL)**	**MAPE**	**RMSE difference vs. proposed model (mg/dL)**
**Proposed model (all modalities)**	**21.04 ± 8.32**	**15.98 ± 7.19**	**0.15 ± 0.05**	-
w/o EDA	25.21 ± 9.04	19.23 ± 8.02	0.18 ± 0.06	+4.17
w/o PPG	26.86 ± 9.51	20.75 ± 8.46	0.19 ± 0.07	+5.82
w/o STEMP	23.39 ± 8.76	17.81 ± 7.63	0.16 ± 0.05	+2.35
w/o ACC	29.47 ± 10.12	22.89 ± 8.97	0.20 ± 0.08	+8.43
w/o EDA + PPG	30.17 ± 10.24	23.46 ± 9.11	0.21 ± 0.08	+9.13
w/o STEMP + ACC	28.65 ± 9.88	21.92 ± 8.74	0.19 ± 0.07	+7.61
w/o EDA + STEMP	26.54 ± 9.42	20.38 ± 8.39	0.19 ± 0.07	+5.50
w/o PPG + ACC	31.29 ± 10.57	24.15 ± 9.32	0.22 ± 0.09	+10.25

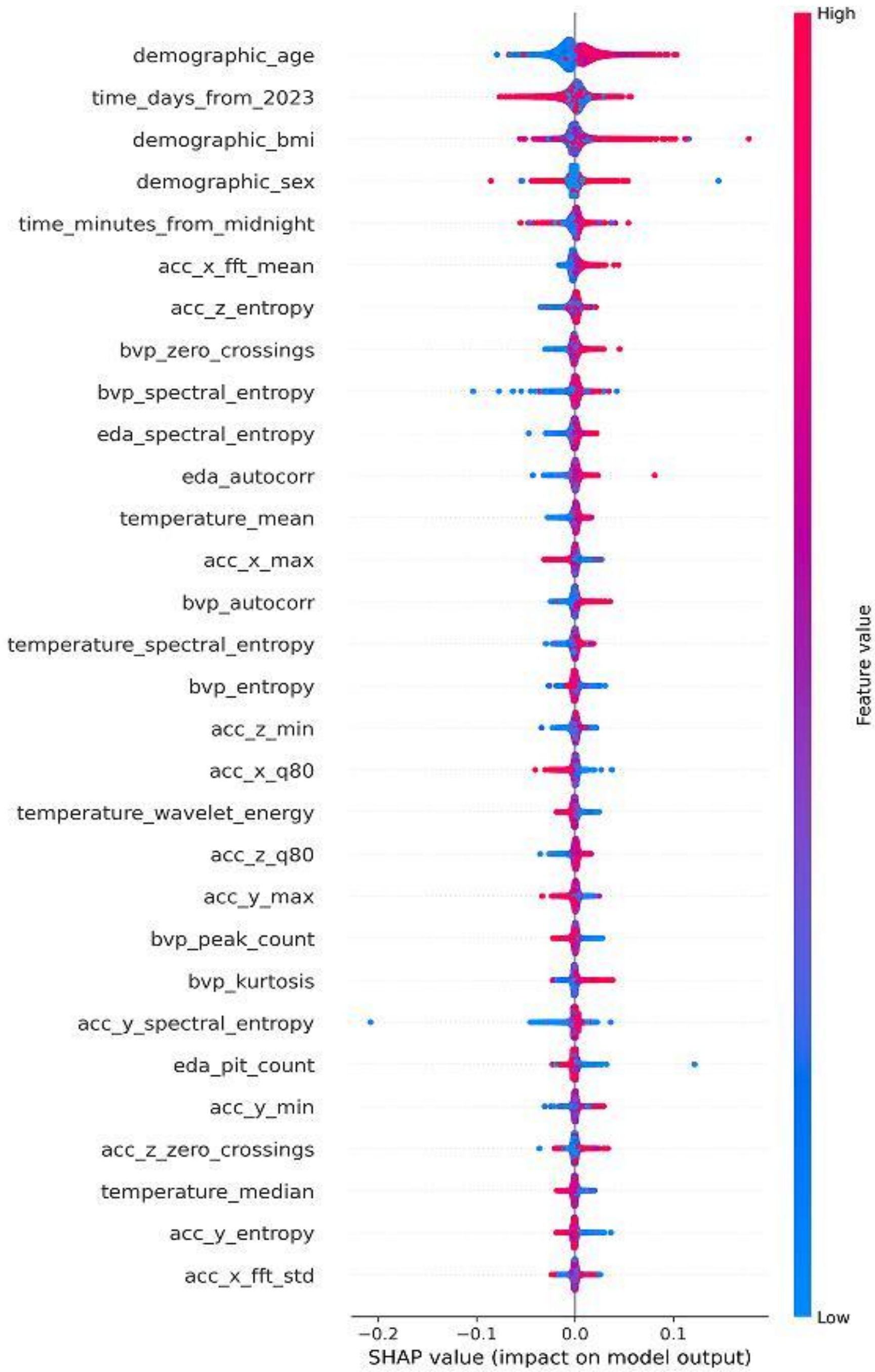

Supplementary Figure S1: SHAP beeswarm plot of the top 30 most influential features for glucose prediction. Each point represents a single sample's SHAP value, which quantifies the feature's impact on the model's glucose prediction output. The color scale indicates feature value (blue = low, red = high). Features are ranked by the magnitude of their average SHAP value (top = most influential). This plot confirms that wearable sensor features (BVP, ACC, EDA, TEMP) are as critical patterns influencing model performance as demographic and temporal attributes.
